# Assessment of stress hyperglycemia ratio to predict mortality in critically ill patients with sepsis: a retrospective cohort study from the MIMIC-IV database

**DOI:** 10.3389/fendo.2025.1496696

**Published:** 2025-03-28

**Authors:** Dong Xia, Xing Luo, Youfeng Zhu, Jianqiu Zhu, Yingqiu Xie

**Affiliations:** Department of Intensive Care Unit, Guangzhou Red Cross Hospital, Jinan University, Guangzhou, Guangdong, China

**Keywords:** stress hyperglycemia ratio, sepsis, mortality, restricted cubic spline analysis, MIMIC-IV

## Abstract

**Introduction:**

The stress hyperglycemia ratio (SHR) is a new insulin resistance assessment tool for patients, which has been linked to clinical adverse events. We aimed to explore the SHR–mortality relationship in critically ill patients with sepsis.

**Methods:**

Patients diagnosed with sepsis, along with blood glucose and hemoglobin A1c levels measured within 24 hours of admission, were retrospectively included in the analysis from the MIMIC-IV database between 2008 to 2019. Patients were stratified into quartile groups (quartile 1 (Q1) to quartile 4 (Q4)) according to SHR level, with 28-day mortality as the primary outcome. The SHR and short term mortality association in patients with sepsis was investigated via Cox regression and Kaplan−Meier analyses. The robustness of the results was verified via multivariate adjustments, multicollinearity, least absolute shrinkage and selection operator (LASSO), and the Boruta algorithm method. The complex relationships among the SHR, short-term mortality were estimated via restricted cubic spline (RCS) analyses.

**Results:**

2407 sepsis patients were involved, with a median age of 67 years, and 59.5% were male. Overall, 28-day, 60-day and 90-day mortality were 17.49% (n=421), 21.31% (n=513) and 23.89% (n=575), respectively. After adjusting confounding variables, the SHR was associated with greater short-term mortality, including 28-day (hazard ratio (HR)=1.14, 95% confidence interval (CI)=1.04-1.24, p=0.005; Q4 vs. Q1 (reference group), HR=1.41, 95% CI=1.06-1.87, p=0.017, p_trend=0.005), 60-day (HR=1.12, 95% CI=1.02-1.70, p=0.015; Q4 vs. Q1, HR=1.32, 95% CI=1.02-1.72, p=0.037, p_trend=0.021) and 90-day (HR=1.11, 95% CI=1.02-1.22, p=0.019; Q4 vs. Q1, HR=1.32, 95% CI=1.03–1.68, p=0.027, p_trend=0.017) mortality. Furthermore, the RCS analysis revealed a quasi U-shaped relationship with regards to SHR and short-term mortality in sepsis. The mortality rate increased with a SHR value larger or smaller than 0.9.

**Conclusions:**

Our research revealed that SHR could serve as a novel indicator for predicting short-term mortality in sepsis patients. SHR demonstrated a quasi U-shaped relationship with short-term mortality in sepsis.

## Introduction

Sepsis is a life-threatening organ dysfunction, which is caused by the dysregulation of host response to infectious pathogens (1). It is a complex condition and one of the most commonly encountered diseases in intensive care units (ICUs) (2, 3). Moreover, the healthcare burden associated with sepsis may be significantly greater than that previously reported, even potentially exceeding that associated with coronary heart disease or stroke (4–6). Although great progress has been made in treating sepsis, the persistent high incidence and elevated mortality rates remain cause concerns.

Stress hyperglycemia during severe illness is characterized by a significant elevation in blood glucose levels in response to critical circumstances (7). It is defined as the blood glucose level greater than 11 mmol/L at admission, regardless of diabetes diagnosis (7, 8). Meanwhile, stress hyperglycemia is considered to be an important indicator of disease severity in patients with sepsis, and is associated with increased mortality in the ICU (9). However, acute stress and severe disturbances in glucose metabolism can lead to excessive activation of sympathetic pathways, inhibition of insulin and promotion of glycolysis, which further leads to overactivity of glucose-mediated proinflammatory pathways (8–10). Furthermore, hyperglycemia exacerbates inflammation by triggering the excessive release of cytokines, resulting in severe inflammatory storms and high mortality in sepsis patients (11).

However, critically ill patients often experience hyperglycemia as a result of a combination of acute stress and underlying chronic glycemic issues rather than solely a sudden increase in glucose levels (11, 12). To reduce the influence of baseline blood glucose levels, researchers have introduced glycated hemoglobin (HbA1c) as an adjustment factor for detecting stress hyperglycemia. Consequently, researchers have proposed the stress hyperglycemia ratio (SHR), which is calculated by dividing the admission blood glucose level by the HbA1c value, as an innovative marker to accurately represent acute hyperglycemia in patients (12). Moreover, several studies have demonstrated a connection between a higher SHR and the occurrence of adverse outcomes in various diseases, including myocardial infarction (MI), heart failure and stroke (13–15). However, few studies have focused on sepsis. Therefore, the objective of this study was to explore the relationship between SHR and mortality in critically ill patients with sepsis.

## Methods

### Sources of data

In this analysis, we utilized data extracted from the Medical Information Mart for Intensive Care IV v2.2 (MIMIC-IV v2.2), a public health record dataset containing clinical information on over 190,000 admitted patients, 450,000 hospitalized patients and 73,181 ICU patients from 2008 to 2019 at the Beth Israel Deaconess Medical Center in Boston, Massachusetts, USA (16). MIMIC-IV v2.2 is updated from MIMIC-IV v2.0, with data enhancements and table reconstructions. One of the authors (Youfeng Zhu) obtained a license to access the database (agreement date: January 4, 2021). The study was prepared in accordance with the Strengthening the Reporting of Observational Studies in Epidemiology (STROBE) statement (17).

### Inclusion and exclusion criteria

Adult patients meeting the sepsis-3.0 diagnosis criteria were involved in this study. Our study only included data for patients’ first admission to the ICU. The exclusion criteria were as follows: individuals under 18 years of age, individuals with repeated ICU admissions (only the first ICU admission data were involved), patients with missing data on serum glucose and HbA1c levels within 24 hours of admission, and those with ICU stays of less than 24 hours.

### Outcome and definitions

The primary outcome in the study was 28-day mortality. The secondary outcomes were 60-day mortality and 90-day mortality. In our study, short-term mortality was defined as 28-day, 60-day, and 90-day mortality. The calculation of the SHR was derived from the following formula (12):

SHR = (admission glucose in mmol/L)/(1.59 * HbA1c [%] - 2.59).

### Data extraction

The process of extracting data was carried out via Navicat Premium (version 17.0.12) and Structured Query Language (SQL) (18). A comprehensive set of data was collected for each patient upon admission, covering various aspects, including (1) patient characteristics, such as age, sex, and weight; (2) vital signs, including mean arterial pressure (MAP), respiratory rate (RR) and heart rate (HR); (3) laboratory indicators, including hemoglobin (Hb), creatinine (Cr), white blood cell level (WBC), blood glucose, platelet count (PLT), blood lactate (LAC), albumin (ALB) and other laboratory serum electrolyte records (sodium, potassium, phosphate, chloride, calcium, and magnesium); (4) pre-ICU comorbidities, including diabetes, hypertension, MI, malignant cancer, chronic pulmonary disease, chronic kidney disease (CKD), severe liver disease, and cerebrovascular disease; (5) severity of organ dysfunction, including Simplified Acute Physiology Score II (SAPSII), Charlson comorbidity index, Sequential Organ Failure Assessment (SOFA), and the Oxford Acute Severity of Illness Score (OASIS); (6) treatment during hospitalization, including the use of vasoactive drugs and continuous renal replacement therapy (CRRT); and (7) hospital and ICU stay data, including 28-day, 60-day and 90-day mortality data and the duration of ICU and hospital stays. All data were obtained within the first 24 hours after the patient was admitted to the ICU. Any variables that had missing values larger than 30% were omitted from the subsequent analysis. For variables that had missing values less than 30%, the missing data were addressed through the application of the multiple imputation method.

### Statistical analysis

Continuous variables were examined to determine if they followed a normal distribution. Student’s t test or one-way ANOVA was used to analyze normally distributed data, which are presented as the means ± standard deviations (SDs). In the case of nonnormally distributed data, the Kruskal−Wallis test or the Mann−Whitney U test was utilized, with results presented as medians with interquartile ranges (IQRs). The analyses of categorical variables were by Fisher’s exact test or the chi-square test, with values shown as numbers and percentages.

The patients were divided into four groups according to the quartile of SHR: quartile 1, with SHR<0.91; quartile 2, with 0.91≤SHR<1.13; quartile 3, with 1.13≤SHR<1.44; and quartile 4, with SHR≥1.44. The reference group for the SHR was the lowest quartile group (quartile 1).

To investigate the relationship between the SHR and short-term mortality, we performed multivariable Cox proportional hazards regression models to evaluate the hazard ratio (HR) and the 95% confidence interval (CI). And Model I was not adjusted for any confounding variables. Model II was adjusted for confounding variables that were statistically significant in the Cox regression analysis for mortality. Additionally, the variance inflation factor (VIF) was utilized to evaluate multicollinearity among parameters. Any variables with a VIF greater than 5 were eliminated to prevent potential concerns related to multicollinearity.

The Boruta algorithm serves as a valuable tool for identifying key factors within a dataset and is used to assess the importance of variables related to outcomes. Furthermore, least absolute shrinkage and selection operator (LASSO) regression was applied to simplify model complexity and mitigate the bias of overfitting variables. After initial screening of the variables via the Boruta algorithm, subsequently we performed LASSO regression analysis as Model III.

We constructed Kaplan−Meier survival curves to compare the short-term mortality rates among groups, as determined by the log-rank test. By adding the SHR to the illness severity scores (including the Charlson comorbidity index, OASIS, SOFA score and SAPSII score), the area under the operator curve (AUC) was used to explore the capacity of the SHR to predict short-term mortality. Furthermore, subgroup analyses, such as age, sex, diabetes, malignant cancer and chronic kidney disease, were performed to confirm the relationship between the SHR and mortality.

We examined the dose−response relationship between the SHR and mortality in sepsis patients via restricted cubic spline (RCS) analysis. The log-likelihood test was used to assess the nonlinearity of smooth curve fitting. Different node values ranging from 3 to 7 were assessed.

The statistical analyses were performed via SPSS software (version 27.0.1, IBM Corporation, United States), R software (version 4.3.2, R Foundation for Statistical Computing, Austria) and STATA (version 18.0, United States). A p-value < 0.05 was considered to indicate statistical significance.

## Results

### Baseline characteristics

**Figure 1:**
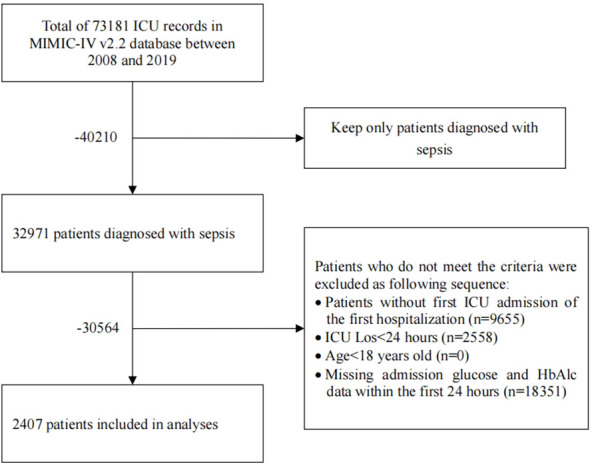
Flow chart of patient’s enrollment.

After screening individuals diagnosed with sepsis in the MIMIC IV database, we identified a specific cohort of 2407 patients who met the predetermined inclusion criteria for this study. A flowchart detailing the selection process for this study is presented in **Figure 1**.

The baseline characteristics of the study population were categorized according to 28-day mortality. The median age of the overall population was 67 (interquartile 56,77) years. Compared with survivors, nonsurvivors tended to be older (66 years (55, 76) vs. 73 years (60, 82), P<0.001) and had a greater proportion of patients with diabetes, cerebrovascular disease, use of vasoactive drugs and CRRT (**Table 1**). Nonsurvivors also presented significantly higher WBC, lactate, potassium, phosphate, and chloride levels (**Table 1**). Additionally, nonsurvivors had higher SOFA, SAPSII, Charlson, OASIS and SHR scores than survivors did (**Table 1**).

**Table 1:** Patients demographics and baseline characteristics.

Variables	Total (n=2407)	Survivors (n = 1986)	Non-survivors (n = 421)	P value	Q1 (n= 595)	Q2 (n= 597)	Q3 (n= 605)	Q4 (n= 610)	P value
Demographics and characteristics									
Age,year,median (IQR)	67 (56, 77)	66 (55, 76)	73 (60, 82)	<0.001	68 (57, 77)	68 (57, 78)	68 (55, 79)	66 (55, 75)	0.022
Weight,median (IQR)	82 (69, 99)	83 (69, 100)	79 (67, 95)	<0.001	82 (66, 100)	81 (69, 100)	81 (69, 97)	83 (70, 100)	0.280
Male,%	1,433 (59.5%)	1,202(60.5%)	231(54.9%)	0.032	350(58.8%)	374(62.6%)	336(55.5%)	373(61.1%)	0.066
Vital Signs									
Respiratory rate,median (IQR)	19 (16, 23)	19 (16, 23)	20 (16, 24)	0.005	19 (15, 22)	19 (16, 23)	20 (16, 24)	20 (16, 24)	0.035
Heart rate, mean (SD)	90 ± 21	90 ± 20	90 ± 22	0.827	86 ± 20	89 ± 21	92 ± 20	94 ± 21	<0.001
MAP,mmHg,mean (SD)	85 ± 19	85 ± 19	85 ± 19	0.882	85 ± 18	85 ± 18	85 ± 19	84 ± 20	0.625
Laboratory findings									
WBC count,×109/L,median (IQR)	12 (9, 16)	11 (8, 16)	13 (10,18)	<0.001	10 (7, 14)	11 (8, 14)	13 (10, 17)	14 (10, 19)	<0.001
Platelet count,×109/L, median (IQR)	199(145, 264)	198(145, 263)	204(146,268)	0.427	206(149, 265)	195(144, 262)	198(145, 264)	197(145, 265)	0.584
Hemoglobin, g/dL, mean (SD)	11.35± 2.34	11.36± 2.30	11.33± 2.51	0.811	11.32± 2.28	11.41± 2.24	11.43± 2.30	11.25± 2.51	0.509
Creatinine, mg/dL,Median (IQR)	1.10(0.80, 1.60)	1.10(0.80, 1.60)	1.20(0.90, 1.80)	0.009	1.00(0.80, 1.50)	1.00(0.80, 1.40)	1.00(0.80, 1.50)	1.30(0.90, 1.90)	<0.001
Blood glucose, mg/dL, median (IQR)	144(112, 206)	143(112, 203)	148(116, 219)	0.082	103(89, 125)	122(109, 144)	153(134, 181)	245(188, 327)	<0.001
Lactate, mmol/L,Median (IQR)	1.62(1.20, 2.60)	1.60(1.10, 2.50)	1.80(1.30, 2.90)	<0.001	1.40(1.00, 2.10)	1.50(1.10, 2.20)	1.70(1.20, 2.60)	2.30(1.50, 3.60)	<0.001
Sodium,mEq/L,median (IQR)	139(135, 141)	139(135, 141)	139(136, 142)	0.056	140(136, 142)	139(136, 142)	139(135, 141)	137(134, 141)	<0.001
Potassium,mEq/L,Median (IQR)	4.10(3.70, 4.60)	4.10(3.70, 4.60)	4.20(3.80, 4.70)	0.013	4.10(3.70, 4.60)	4.10(3.70, 4.50)	4.10(3.70, 4.50)	4.20(3.80, 4.80)	<0.001
Phosphate,mEq/L,Median (IQR)	3.50(2.90, 4.30)	3.50(2.80, 4.30)	3.70(2.90, 4.72)	0.003	3.50(2.80, 4.13)	3.50(2.80, 4.10)	3.40(2.80, 4.20)	3.80(3.00, 4.90)	<0.001
Chloride,mEq/L,Median (IQR)	103(100, 107)	103(99, 107)	105(100, 108)	0.008	104(101, 107)	104(100, 107)	103(100, 107)	102(98, 107)	<0.001
Calcium,mg/dL,Median (IQR)	8.40(7.90, 8.90)	8.40(7.80, 8.90)	8.40(7.90, 8.90)	0.924	8.40(7.90, 8.90)	8.40(7.90, 8.90)	8.40(7.80, 8.90)	8.30(7.70, 8.90)	0.012
Magnesium,mg/dL, median (IQR)	1.90(1.70, 2.20)	1.90(1.70, 2.20)	1.90(1.70, 2.20)	0.797	2.00(1.75, 2.20)	1.90(1.80, 2.10)	1.90(1.70, 2.20)	1.90(1.70, 2.20)	0.592
Albumin, g/dL,mean (SD)	3.18± 0.63	3.19± 0.62	3.10± 0.66	0.011	3.24± 0.63	3.22± 0.62	3.17± 0.62	3.08± 0.64	<0.001
Comorbidity									
Myocardial infarct, no. (%)	659(27.4%)	537(27.0%)	122(29.0%)	0.418	166(27.9%)	135(22.6%)	163(26.9%)	195(32.0%)	0.004
Chronic kidney disease, no. (%)	485(20.1%)	392(19.7%)	93(22.1%)	0.274	122 (20.5%)	122 (20.4%)	100(16.5%)	141(23.1%)	0.039
Chronic pulmonary disease, no. (%)	595(24.7%)	502(25.3%)	93(22.1%)	0.169	172(28.9%)	132 (22.1%)	134(22.1%)	157(27.7%)	0.016
Sever liver disease, no.(%)	130 (5.4%)	102 (5.1%)	28 (6.7%)	0.212	15 (2.5%)	24 (4.0%)	35 (5.8%)	56 (9.2%)	<0.001
Cerebrovascular disease, no. (%)	892(37.1%)	672(33.8%)	220(52.3%)	<0.001	212 (35.6%)	259 (43.4%)	248(41.0%)	173(28.4%)	<0.001
Malignant cancer, no.(%)	195 (8.1%)	152 (7.7%)	43 (10.2%)	0.080	54 (9.1%)	43 (7.2%)	51 (8.4%)	47 (7.7%)	0.654
Hypertension, no.(%)	1,086 (45.1%)	906(45.6%)	180(42.8%)	0.283	268 (45.0%)	275 (46.1%)	301(49.8%)	242(39.7%)	0.005
Diabetes, no.(%)	1,008 (41.9%)	859(43.3%)	149(35.4%)	0.003	276 (46.4%)	196 (32.8%)	228(37.7%)	308(50.5%)	<0.001
Organ dysfunction									
SOFA, median (IQR)	5.0(3.0, 7.0)	5.0(3.0, 7.0)	6.0(4.0, 8.0)	<0.001	4.0(3.0, 7.0)	4.0(2.0, 7.0)	5.0(3.0, 7.0)	6.0(4.0, 9.0)	<0.001
SAPSII, median (IQR)	37 (30, 45)	36 (29,44)	42 (35, 51)	<0.001	36 (28, 45)	35 (29, 43)	37 (30, 43)	40 (33, 49)	<0.001
Charslon, median (IQR)	5.00(3.00, 8.00)	5.00(3.00, 7.00)	7.00(4.00, 8.00)	<0.001	6.00(4.00, 8.00)	5.00(3.00, 7.00)	5.00(3.00, 8.00)	6.00(4.00, 8.00)	0.431
OASIS, mean (SD)	34 ± 8	33 ± 8	37 ± 8	<0.001	33 ± 8	33 ± 8	34 ± 8	36 ± 8	<0.001
Treatment during hospitalization									
CRRT, no. (%)	148 (6.1%)	93 (4.7%)	55 (13.1%)	<0.001	27 (4.5%)	29 (4.9%)	36 (6.0%)	56 (9.2%)	0.003
Vasoactive drugs, no. (%)	962(40.0%)	757(38.1%)	205(48.7%)	<0.001	220 (37.0%)	194 (32.5%)	232(38.3%)	316(51.8%)	<0.001
Outcomes									
SHR (Quartile)				0.002	–				
Q1, no.(%)	595 (24.7%)	511 (25.7%)	84 (20.0%)		–				
Q2, no.(%)	597 (24.8%)	506 (25.5%)	91 (21.6%)		–				
Q3, no.(%)	605 (25.1%)	492 (24.8%)	113 (26.8%)		–				
Q4, no.(%)	610(25.3%)	477(24.0%)	133(31.6%)		–				
ICU stay, days, median (IQR)	4.5(2.4, 8.7)	4.2(2.3, 8.3)	5.6(3.0, 9.6)	<0.001	3.9(2.1, 7.5)	4.5(2.4, 8.5)	5.1(2.6, 9.7)	4.5(2.7, 8.8)	<0.001
Hospital stay, days, median (IQR)	12 (7, 21)	13 (8, 22)	9 (5, 15)	<0.001	11 (7, 19)	13 (8, 21)	14 (8, 23)	12 (7, 21)	<0.001

IQR, Interquartile Range; SD, Standard Deviation; MAP, Mean Artery Pressure; WBC, White Blood Cell count; ALB, Albumin; SOFA, Sequential Organ Failure Assessment; SAPSII, Simplified Acute Physiology Score II; OASIS, Oxford acute severity of illness score; CRRT, Continuous Renal Replacement Therapy; SHR, Stress Hyperglycemia Ratio; SHR: Q1,Quartile 1 (<0.91); Q2, Quartile 2 (0.91–1.13); Q3, Quartile 3 (1.13–1.44); Q4, Quartile 4 (≥1.44).

Vasoactive drugs agents were defined as any use of norepinephrine, epinephrine, dopamine, phenylephrine, milrinone, and dobutamine within the first two days of ICU admission.

### Relationship between the SHR and short-term mortality

**Figure 2:**
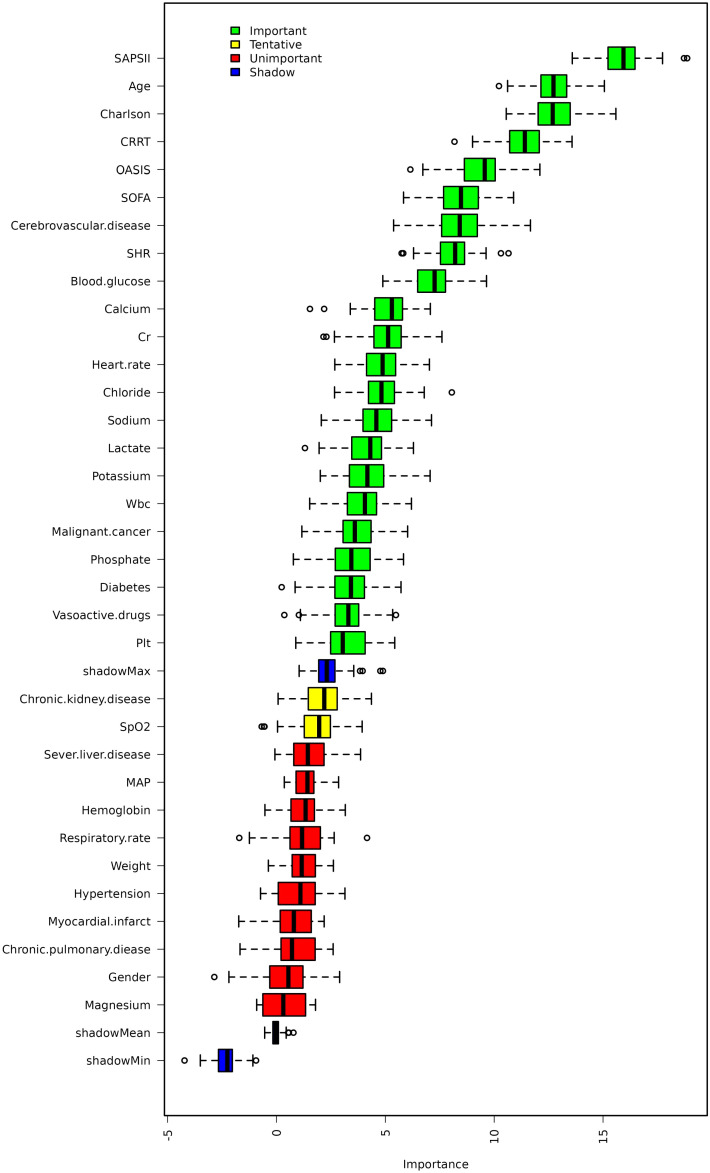
Feature selection based on the Boruta algorithm.

**Figure 3:**
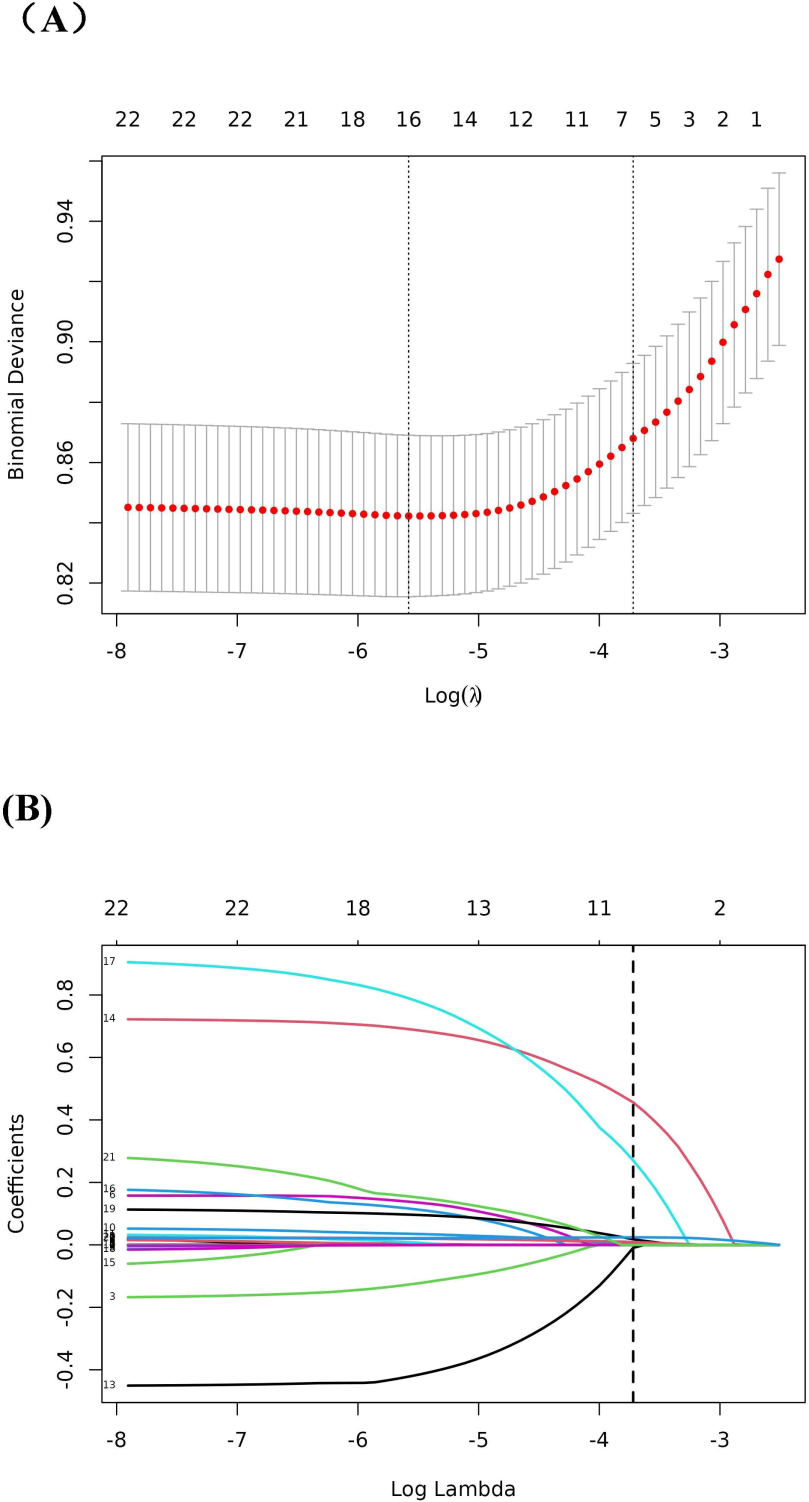
LASSO regression analysis for the screening of predictor variables. **(A)** Tuning parameter (λ) selection by cross-validation method; **(B)** Plot of the Lasso coefficient profiles.

The patients were divided into four groups according to the quartile of SHR: quartile 1 [N =595], with SHR<0.91; quartile 2 [N =597], with 0.91≤SHR<1.13; quartile 3 [N =605], with 1.13≤SHR<1.44; and quartile 4 [N =610], with SHR≥1.44). During the follow-up period of 28 days, 60 days and 90 days, 421, 513 and 575 deaths were recorded, respectively. Cox regression analysis revealed a significant relationships between the SHR and 28-day, 60-day and 90-day mortality (**Table 2**). This relationship was identified in both unadjusted Model I and fully adjusted Model II and Model III (**Table 2**). Model II was adjusted for confounding parameters that were statistically significant in the Cox regression analysis for mortality (**Supplementary File Table S1**). Model III was adjusted for variables that selected through the Boruta and LASSO analysis (**Figures 2**, **3**).

**Table 2:** Association between SHR and short-term mortality in patients with sepsis.

Outcomes	Model I HR(95% CI), P	Model II HR(95% CI), P	Model III HR(95% CI), P
28-days mortality			
SHR (continuous variable) ^a^	1.18(1.09,1.28), <0.001	1.14(1.04,1.25), 0.006	1.14(1.04,1.24), 0.005
SHR (quartile variable)			
Q1	1.0(Ref)	1.0(Ref)	1.0(Ref)
Q2	1.09(0.81,1.47), 0.558	1.01(0.75,1.35), 0.973	0.99(0.74,1.34), 0.969
Q3	1.36(1.03,1.81), 0.032	1.18(0.89,1.57), 0.255	1.17(0.88,1.56), 0.278
Q4	1.65(1.26,2.17), <0.001	1.39(1.04,1.85), 0.024	1.41(1.06,1.87), 0.017
P for trend	<0.001	0.012	0.005
60-days mortality			
SHR (continuous variable)^a^	1.16(1.07,1.26), <0.001	1.12(1.02,1.23), 0.017	1.12(1.02,1.22), 0.015
SHR (quartile variable)			
Q1	1.0(Ref)	1.0(Ref)	1.0(Ref)
Q2	1.12(0.86,1.47), 0.383	1.03(0.79,1.34), 0.830	1.02(0.79,1.34), 0.859
Q3	1.39(1.08,1.79), 0.011	1.22(0.94,1.58), 0.133	1.22(0.94,1.57), 0.131
Q4	1.52(1.19,1.95), <0.001	1.31(1.01,1.70), 0.044	1.32(1.02,1.70), 0.037
P for trend	<0.001	0.020	0.021
90-days mortality			
SHR (continuous variable)^a^	1.16(1.07,1.25), <0.001	1.11(1.02,1.22), 0.022	1.11(1.02,1.22), 0.019
SHR (quartile variable)			
Q1	1.0(Ref)	1.0(Ref)	1.0(Ref)
Q2	1.15(0.90,1.48), 0.273	1.04(0.81,1.34), 0.739	1.05(0.81,1.34), 0.723
Q3	1.40(1.10,1.78), 0.006	1.23(0.96,1.57), 0.099	1.24(0.97,1.58), 0.087
Q4	1.52(1.20,1.93), <0.001	1.31(1.02,1.68), 0.034	1.32(1.03,1.68), 0.027
P for trend	<0.001	0.015	0.017

SHR, Stress Hyperglycemia Ratio; SHR: Q1,Quartile 1 (<0.91); Q2, Quartile 2 (0.91–1.13); Q3, Quartile 3 (1.13–1.44); Q4, Quartile 4 (≥1.44); HR, Hazard Ratio; CI, Confidence Interval; Ref, reference. WBC, White Blood Cell count; ALB, Albumin; SOFA, Sequential Organ Failure Assessment; SAPSII, Simplified Acute Physiology Score II; OASIS, Oxford acute severity of illness score; CRRT, Continuous Renal Replacement Therapy; Vasoactive drugs agents were defined as any use of norepinephrine, epinephrine, dopamine, phenylephrine, milrinone, and dobutamine within the first two days of ICU admission.

a: Cox proportional hazards regression analysis with SHR as a continuous variable to assess the relationship between SHR and mortality.

Model I: adjusted for none.

Model II: Adjust for variables that were statistically significant in the Cox proportional hazards regression analysis for mortality:Age; Sex; Weight; Respiratory rate; WBC; ALB; Lactate; Potassium; Potassium; Cerebrovascular disease; Diabetes; CRRT; Vasoactive drugs; SOFA; SAPSII; OASIS; Charlson.

Model III: Adjust for variables that selected through the Boruta and LASSO analysis: WBC; Diabetes; Cerebrovascular disease; CRRT; SAPSII; OASIS; Charlson.

When SHR was analyzed as a continuous variable, as shown in Model III, a larger SHR was correlated with an increased short-term mortality, including 28-day mortality (HR=1.14, 95% CI=1.04-1.24, p=0.005), 60-day mortality (HR=1.12, 95% CI 1.02-1.70, p=0.015) and 90-day mortality (HR=1.11, 95% CI 1.02-1.22, p=0.019) (**Table 2**). When the SHR was analyzed as a categorical parameter, with the lowest SHR group (Q1) as the reference, the incidence of short-term mortality increased with increasing SHR values among the different groups in Model III (28-day mortality, P for trend 0.005; 60-day mortality, P for trend 0.021; 90-day mortality, P for trend 0.017).

**Figure 4:**
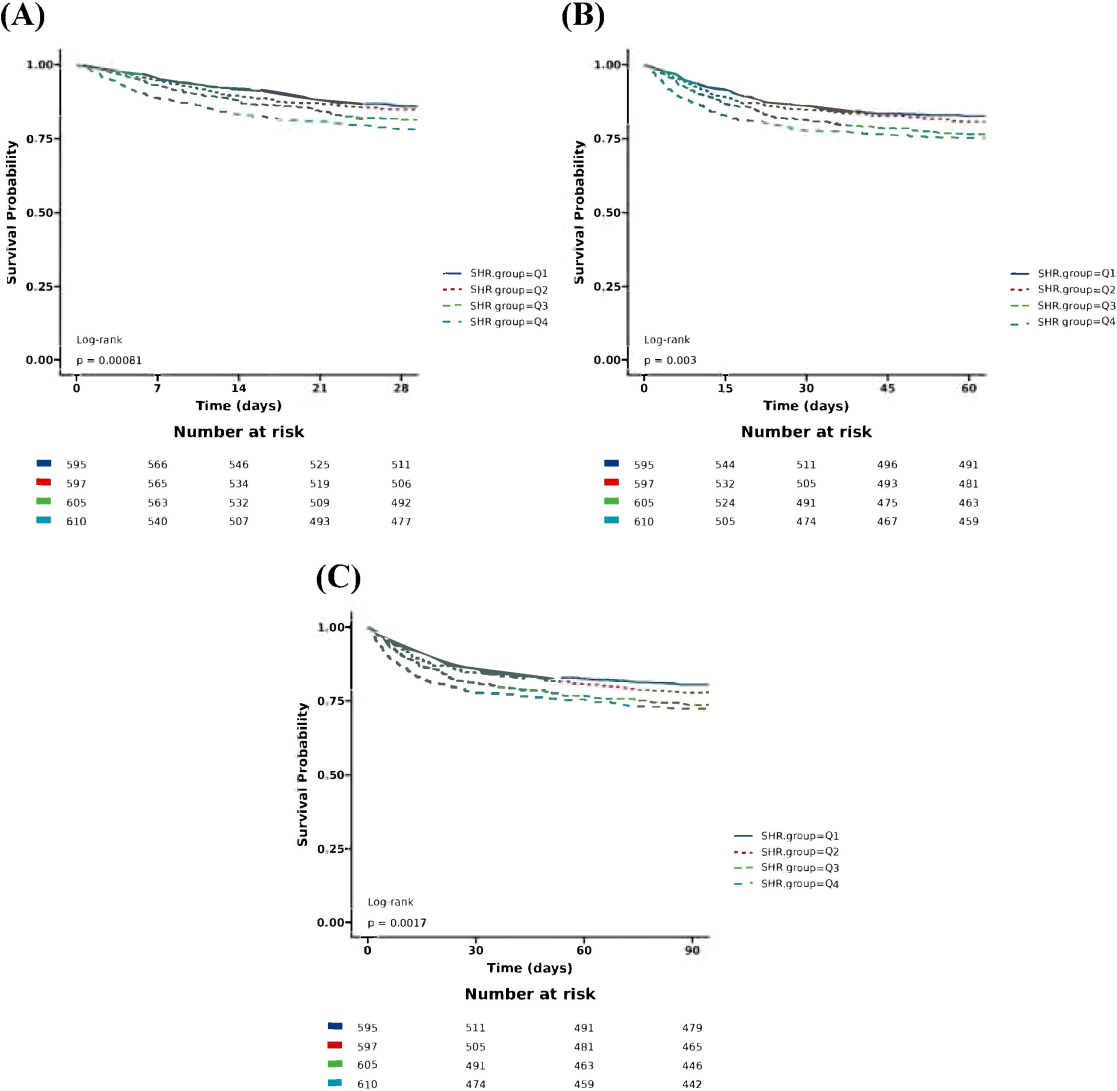
Kaplan–Meier survival analysis for short-term mortality with SHR category. **(A)** Kaplan-Meier survival analysis for 28-day mortality with SHR category; **(B)** Kaplan-Meier survival analysis for 60-day mortality with SHR category; **(C)** Kaplan-Meier survival analysis for 90-day mortality with SHR category. SHR, Stress Hyperglycemia Ratio; SHR: Ql, Quartile 1 (<0.91); Quartile 2 (0.91-1.13); Quartile 3 (1.13-1.44 ); Quartile 4(1.44).

The Boruta algorithm also revealed that the SHR plays a crucial role in the short-term mortality of sepsis patients (**Figure 2**). The RCS showed a quasi U-shaped association between the SHR and short-term mortality (P value <0.05; **Supplementary Figure S1**). The mortality rate increased with a SHR value larger or smaller than 0.9 (**Supplementary Figure S1**). In the initial downward slope on the left side of the U-shape curve, a smaller SHR value means a higher mortality rate which was mainly caused by hypoglycemia (**Supplementary Figure S1**). Survival analysis revealed that the SHR was correlated with a rise of 28-day, 60-day, and 90-day mortality (Kaplan–Meier, log-rank P < 0.001; **Figure 4**). We investigated whether SHR could be integrated with traditional clinical scores, such as SOFA score, Charlson score, SAPSII score, OASIS score, to provide a more comprehensive predictive value (**Table 3**). We performed ROC analysis to assess the area under the curve (AUC) for both the traditional clinical score alone (Model I) and the traditional clinical scores plus SHR (Model II) (**Table 3**). The results showed that the AUC of Charlson score plus SHR was larger than Charlson score alone (28-day mortality (Model I 0.608 (0.578 to 0.638) vs Model II 0.635 (0.596 to 0.654), p=0.006), 60-day mortality (Model I 0.630 (0.603 to 0.658) vs Model II 0.639 (0.612 to 0.666), p=0.024), and 90-day mortality (Model I 0.647(0.621 to 0.673) vs Model II 0.655 (0.630 to 0.681), p=0.011)). For SOFA score plus SHR, SAPSII score plus SHR and OASIS score plus SHR, though the results did not achieve a statistical significance, we observed an increasing trend in the predictive performance of AUC (**Table 3**).

**Table 3:** Comparison of the area under the receiver (AUC) operating characteristic curves of the models for SHR in predicting short-term mortality in sepsis.

Categories	Model I	Model II	P value (Model 2 vs Model 1)
	AUC and 95%CI	AUC and 95%CI	
28-day mortality			
Charlson Score	0.608 (0.578 to 0.638)	0.635 (0.596 to 0.654)	0.006
SOFA Score	0.588 (0.558 to 0.618)	0.597 (0.568 to 0.627)	0.057
OASIS Score	0.631 (0.601 to 0.660)	0.635 (0.606 to 0.664)	0.166
SAPSII Score	0.658 (0.630 to 0.686)	0.660 (0.632 to 0.688)	0.392
60-day mortality			
Charlson Score	0.630 (0.603 to 0.658)	0.639 (0.612 to 0.666)	0.024
SOFA Score	0.588 (0.560 to 0.616)	0.594 (0.566 to 0.621)	0.082
OASIS Score	0.632 (0.605 to 0.659)	0.634 (0.607 to 0.661)	0.318
SAPSII Score	0.663 (0.637 to 0.689)	0.664 (0.638 to 0.689)	0.641
90-day mortality			
Charlson Score	0.647 (0.621 to 0.673)	0.655 (0.630 to 0.681)	0.011
SOFA Score	0.588 (0.562 to 0.615)	0.593 (0.567 to 0.620)	0.096
OASIS Score	0.634 (0.608 to 0.659)	0.635 (0.609 to 0.661)	0.444
SAPSII Score	0.668 (0.644 to 0.692)	0.668 (0.644 to 0.693)	0.829

SHR, stress hyperglycemia ratio; ICU, intensive care unit; SOFA, Sequential Organ Failure Assessment; SAPSII, Simplified Acute Physiology Score II; OASIS, Oxford acute severity of illness score; AUC, area under the curve; CI, confidence interval.

Model I: traditional clinical score alone.

Model II: traditional clinical score alone+SHR.

### Subgroup analysis

**Figure 5:**
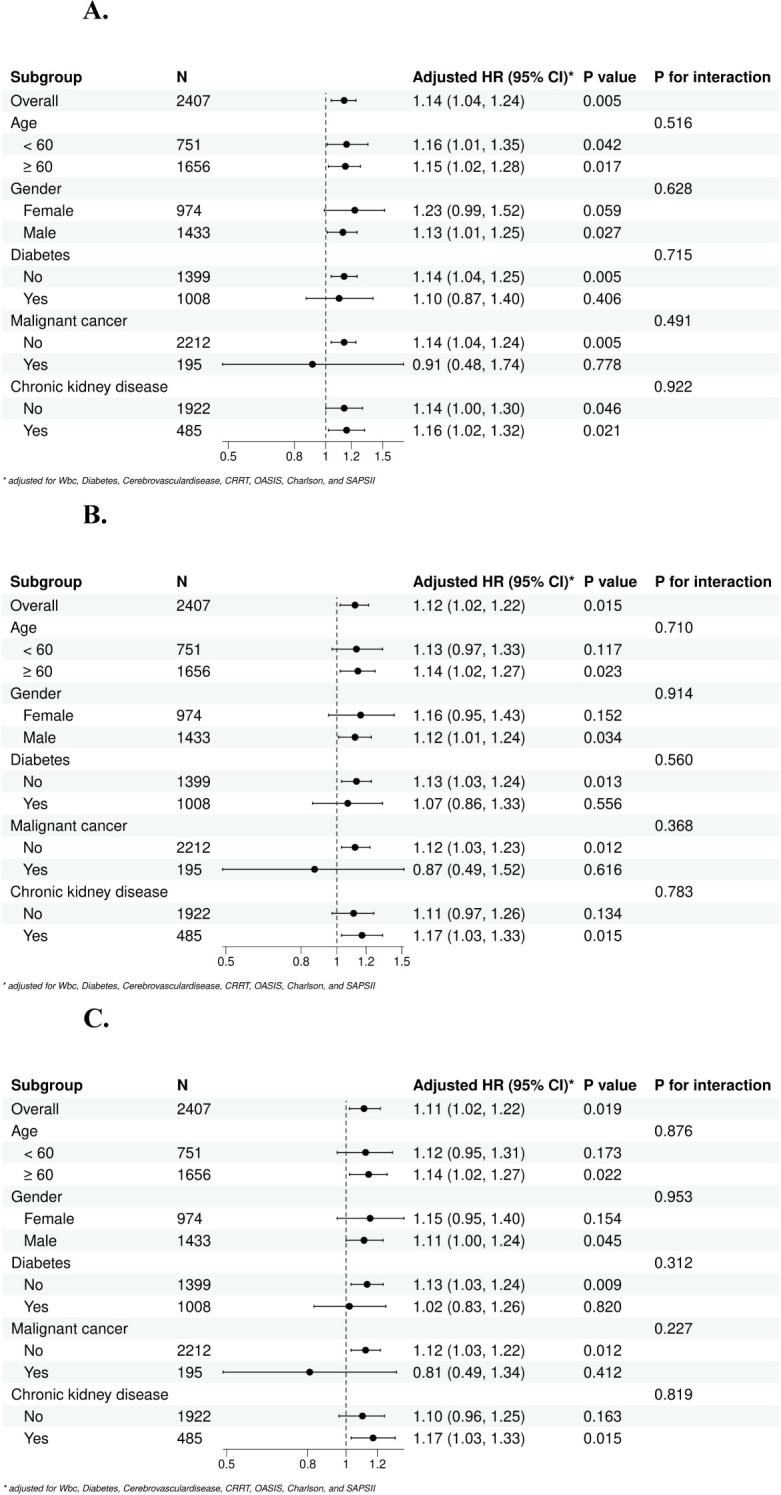
Subgroup forest plot for short-term [**(A)** 28-day, **(B)** 60-day, **(C)** 90-day] all-cause mortality. **(A)** Subgroup analysis for 28-day all-cause mortality; **(B)** Subgroup analysis for 60-day all-cause mortality; **(C)** Subgroup analysis for 90-day all-cause mortality.

To further explore the relationship between the SHR and mortality in sepsis patients, we performed subgroup analysis by stratifying the patients according to sex, age, diabetes, malignant cancer status, chronic kidney disease status. The p values for interactions in all subgroups were greater than 0.05, which indicated that these factors did not significantly influence the relationship between the SHR and mortality in sepsis patients (**Figure 5**).

## Discussion

In our study, we explored the SHR and mortality relationship in critically ill patients with sepsis, and demonstrated that a quasi U-shaped association between the SHR and short-term mortality was observed. The mortality rate increased with a SHR value larger or smaller than 0.9. The risk of 28-day, 60-day and 90-day mortality in sepsis patients increased by 14%, 12%, and 11%, respectively, for each one-unit increase in the SHR. In the highest quartile group (Q4), compared with that in the lowest quartile group (Q1), the 28-day, 60-day and 90-day mortality in patients with sepsis increased by 41%, 32% and 32%, respectively.

It is well known that poor glycemic control during hospitalization is associated with worse outcomes in sepsis (19–22). Our result was not conflicting with the effect of hypoglycemia on mortality. Our study showed a quasi U-shape between SHR and short term mortality in sepsis patients. The mortality rate increased with a SHR value larger or smaller than 0.9 (**Supplementary Figure S1**). In the initial downward slope on the left side of the U-shape curve, a smaller SHR value means a higher mortality rate which was mainly caused by hypoglycemia (**Supplementary Figure S1**). In light of this point, we recommend that clinicians should not only focus on patients with high SHR values but also pay close attention to those with SHR values lower than 0.9. These patients may be at elevated risk of mortality due to the effects of hypoglycemia.

In critically ill patients with sepsis, previous studies revealed that stress hyperglycemia can cause inflammatory responses and oxidative stress, exacerbating the inflammatory storm and increasing the risk of multiple organ dysfunction syndrome (23). The guidelines of survival sepsis campaign and multiple studies have indicated that maintaining blood glucose levels between 80 and 100 mg/dl through continuous insulin infusion can lead to a decrease in ICU mortality among critically ill patients (24, 25). Recent research has called for targeted efforts to identify individuals at increased risk of harm from high blood glucose levels, with the potential to reduce both inflammation and mortality rates. Therefore, the SHR may be an innovative marker of prognosis in sepsis patients.

An elevated SHR serves as a marker for a hyperglycemic stress state, irrespective of an individual’s prior blood glucose levels, and has been recognized as a significant risk factor for both short- and long-term prognosis in individuals (26, 27). Yan et al. proposed a strong correlation between the SHR and 28-day mortality in sepsis patients (28). On this basis, we further explored 60- and 90-day mortality by more rigorously adjusting for confounding factors. Subsequent studies have revealed that SHR in acute cardiac disease, kidney disease and trauma are closely linked to mortality in nondiabetic patients (29–31). The subgroup analysis of our study revealed consistent results in sepsis patients with or without preexisting diabetes. These findings indicate that the SHR has potential for use in glucose management in sepsis patients.

In animal models, continuously elevated hyperglycemia is harmful to immune function and activates cytokines, leading to the promotion of oxidative and inflammatory storms (32, 33). In times of hyperglycemic stress, the hypothalamic−pituitary−adrenal axis and the sympathetic−adrenal system become active, leading to increased release of proinflammatory cytokines, which further exacerbates the ongoing inflammatory storm (10, 34, 35). Pathophysiologically, elevated blood glucose levels due to stress can result in increased production of reactive oxygen species in the mitochondria of endothelial cells, potentially leading to impaired endothelial function. Furthermore, stress hyperglycemia might be linked to increased endothelial dysfunction and intravascular coagulation risk, resulting in increased capillary leakage and disseminated intravascular coagulation (DIC), respectively (36, 37). An elevated SHR is indicative of a hyperglycemic stress response characterized by a complex interplay of various hormones, including cytokines, glucocorticoids and catecholamines, all of which collectively contribute to an inflammatory reaction within the body (34–38). Previous studies have shown that high glucose levels can promotes the synthesis and release of IL-6 in monocytes. IL-6 is subsequently released in large quantities, promoting hepatic glucose production, inhibiting insulin release, aggravating the occurrence of hyperglycemia and triggering an inflammatory storm (28, 39).

It is well known that glycemic status in critically ill patients is influenced by numerous factors beyond the solitary admission glucose value, including underlying disease, medications, nutritional status, etc. Hence, our study uses the SHR, which incorporates both the admission blood glucose level and the patient’s glycated hemoglobin (HbA1c) level, to adjust for baseline chronic glycemic control condition and the impact of background blood glucose level. The SHR helps minimize the impact of pre-existing glycemic conditions (such as diabetes or metabolic syndrome) on acute glucose changes and can more accurately reflect acute glycemic dysregulation in critically ill patients (12).

To resolve the influence of underlying diseases on SHR, we included pre-ICU comorbidities in our analysis, such as diabetes, chronic kidney disease and other relevant conditions (**Table 2**, **Figure 5**). These comorbidities are known to influence glycemic level and are adjusted in our multivariable regression models (**Table 2**). Meanwhile, the subgroup analyses revealed that all p-values for interactions in all subgroups were greater than 0.05, which indicated that these factors did not influence the relationship between the SHR and mortality in sepsis patients (**Figure 5**).

As glycemic status is influenced by nutritional status, to resolve the influence of nutritional status on SHR, according to previous study (28), we included weight as a variable in our study. Furthermore, we included blood albumin data as a marker of nutritional status. We conducted a Cox regression analysis and variance inflation factor (VIF) assessment (**Supplementary File Table S2**), ultimately incorporating albumin into Model II to ensure a more robust adjustment for nutritional status. The final results demonstrated that the association between SHR and mortality remained consistent with the original findings, even after adjusting albumin into the analysis (HR=1.14, 95% CI=1.04-1.25, p=0.006; Q4 vs. Q1, HR=1.39, 95% CI 1.04-1.85, p=0.024), 60-day mortality (HR=1.12, 95% CI 1.02-1.23, p=0.017; Q4 vs. Q1, HR=1.31, 95% CI 1.01-1.70, p=0.044) and 90-day mortality (HR=1.11, 95% CI 1.02-1.22, p=0.022; Q4 vs. Q1, HR=1.31, 95% CI: 1.02-1.68, p=0.034; **Table 2**).

### Strengths and limitations

To our knowledge, this is the first large-scale study exploring the relationship between the SHR and short-term mortality in sepsis patients. The SHR can be easily used in clinical and be quickly measured for early recognition of critically ill sepsis patients. There were several limitations in our study. First, this study was a single-center analysis based on observational data extracted from the MIMIC-IV database; Therefore, no causal relationship was demonstrated. With regards to this problem, we used rigorous and multiple statistical methods for decreasing the potential influence of confounders. Second, bias could not be avoided because of missing data and unmeasured variables in this study. Third, the glycemic status of critically ill patients is influenced by various medications that are commonly used in intensive care settings, such as insulin, corticosteroids, and vasoactive drugs. These medications can significantly alter blood glucose levels either directly (e.g., insulin therapy) or indirectly (e.g., steroids inducing hyperglycemia). However, the MIMIC database does not provide a recent medication history prior to admission. As we did not have the detailed medication information on each patient, an analysis with regards to the medications on SHR cannot be performed. For resolving the influence of medications on SHR, we included the clinical scores in our analysis, such as SOFA, which indirectly and partially reflected the effects of medications (e.g., use of insulin or steroids) that may influence glycemic status (40, 41). Fourth, the variability in glycemic management after admission could introduce potential biases, highlighting the need for further studies to explore these factors. Large, prospective investigations are needed to overcome these limitations.

## Conclusion

Our research revealed that SHR could serve as a novel indicator for predicting short-term mortality in sepsis patients. SHR demonstrated a quasi U-shaped relationship with short-term mortality in sepsis.

## Data Availability

The datasets presented in this study can be found in online repositories. The names of the repository/repositories and accession number(s) can be found below: The dataset can be found at https://mimic-iv.mit.edu/.

## Supplementary material

Supplementary Table 1 Cox proportional hazards regression analysis of risk factors for the 28-day mortality in sepsis patients.
