# Supplementary material for: Assessment of stress hyperglycemia ratio to predict mortality in critically ill patients with sepsis: a retrospective cohort study from the MIMIC-IV database
Source: Front Endocrinol (Lausanne). 2025 Mar 28;16:1496696. doi: 10.3389/fendo.2025.1496696 (PMC11985425; doi:10.3389/fendo.2025.1496696)
Supplement: Supplementary Table 1 — Cox proportional hazards regression analysis of risk factors for the 28-day mortality in sepsis patients. [file DataSheet1.docx]

**Supplemental file**

**Table S1. Univariate cox regression analysis of risk factors for the 28-day mortality in sepsis patients.**

| **Variables** | **Univariate model** | |  | **Multivariate model** | |
| --- | --- | --- | --- | --- | --- |
|  | Hazard Ratio  (95% CI) | P value |  | Hazard Ratio  (95% CI) | P value |
| Age | 1.02(1.01,1.02) | <0.001 |  | 1.00(0.99,1.01) | 0.573 |
| Weight | 0.99(0.99,1.00) | 0.002 |  | 1.00(0.99,1.00) | 0.460 |
| Male | 0.81(0.67,0.98) | 0.034 |  | 0.96(0.78,1.18) | 0.712 |
| Respiratory rate | 1.02(1.01,1.04) | 0.003 |  | 1.02(1.00,1.03) | 0.034 |
| Heart rate | 1.00(0.99,1.00) | 0.798 |  | - | - |
| MAP | 1.00(0.99,1.00) | 0.793 |  | - | - |
| WBC count | 1.03(1.02,1.04) | <0.001 |  | 1.02(1.01,1.03) | 0.001 |
| Platelet count | 1.00(1.00,1.00) | 0.595 |  | - | - |
| Hemoglobin | 1.00(0.96,1.04) | 0.981 |  | - | - |
| Creatinine | 0.99(0.94,1.05) | 0.847 |  | - | - |
| Blood glucose | 1.00(1.00,1.00) | 0.276 |  | - | - |
| Lactate | 1.10(1.06,1.14) | <0.001 |  | 1.03(0.98,1.08) | 0.197 |
| Sodium | 1.01(0.99,1.03) | 0.333 |  | - | - |
| Potassium | 1.24(1.11,1.38) | <0.001 |  | 1.15(1.02,1.29) | 0.026 |
| Calcium | 0.99(0.89,1.11) | 0.851 |  | - | - |
| Chloride | 1.01(1.00,1.03) | 0.079 |  | - | - |
| Magnesium | 1.09(0.85,1.38) | 0.502 |  | - | - |
| Phosphate | 1.07(1.03,1.11) | <0.001 |  | 0.99(0.93,1.06) | 0.822 |
| Albumin | 0.83(0.71,0.96) | 0.013 |  | 0.98(0.83,1.15) | 0.765 |
| Myocardial infarct | 1.09(0.88,1.34) | 0.447 |  | - | - |
| Chronic kidney disease | 1.13(0.90,1.42) | 0.292 |  | - | - |
| Chronic pulmonary diease | 0.84(0.67,1.06) | 0.146 |  | - | - |
| Sever liver disease | 1.25(0.85,1.83) | 0.261 |  | - | - |
| Cerebrovascular disease | 1.97(1.63,2.39) | <0.001 |  | 1.94(1.57,2.40) | <0.001 |
| Malignant cancer | 1.30(0.95,1.78) | 0.106 |  | - | - |
| Diabetes | 0.74(0.60,0.90) | 0.003 |  | 0.63(0.51,0.79) | <0.001 |
| Hypertension | 0.89(0.73,1.08) | 0.228 |  | - | - |
| CRRT | 2.61(1.96,3.46) | <0.001 |  | 1.74(1.25,2.43) | <0.001 |
| Vasoactive drugs | 1.51(1.25,1.83) | <0.001 |  | 1.25(0.98,1.58) | 0.068 |
| SOFA | 1.09(1.06,1.12) | <0.001 |  | 0.97(0.93,1.01) | 0.161 |
| SAPSII | 1.04(1.03,1.04) | <0.001 |  | 1.02(1.01,1.03) | <0.001 |
| OASIS | 1.05(1.04,1.07) | <0.001 |  | 1.02(1.00,1.03) | 0.046 |
| Charlson | 1.12(1.08,1.15) | <0.001 |  | 1.06(1.01,1.12) | 0.010 |
| SHR | 1.18(1.09,1.28) | <0.001 |  | 1.14(1.04,1.25) | 0.006 |

MAP, Mean Artery Pressure; WBC, white Blood Cell count; SpO2, pulse oxygen saturation; CRRT, Continuous Renal Replacement Therapy; SOFA, Sequential Organ Failure Assessment; SAPSII, Simplified Acute Physiology Score II; OASIS, Oxford acute severity of illness score; SHR, Stress Hyperglycemia Ratio.

Vasoactive drugs agents were defined as any use of norepinephrine, epinephrine, dopamine, phenylephrine, milrinone, and dobutamine within the first two days of ICU admission.

**Table S2. Variance inflation factor among variables**

| **Variables** | **VIF** |
| --- | --- |
| Age | 2.029885 |
| Gender | 1.152603 |
| Weight | 1.275617 |
| Respiratory rate | 1.048116 |
| WBC | 1.130175 |
| Lactate | 1.165384 |
| Potassium | 1.289506 |
| Phosphate | 1.394557 |
| Albumin | 1.125391 |
| Cerebrovascular disease | 1.196627 |
| Diabetes | 1.201475 |
| CRRT | 1.354138 |
| Vasoactive drugs | 1.500015 |
| SOFA | 2.776570 |
| SAPSII | 3.134437 |
| OASIS | 2.006919 |
| Charlson | 2.107227 |

WBC, White Blood Cell count; CRRT, Continuous Renal Replacement Therapy; SOFA, Sequential Organ Failure Assessment; SAPSII, Simplified Acute Physiology Score II; OASIS, Oxford acute severity of illness score;

Vasoactive drugs agents were defined as any use of norepinephrine, epinephrine, dopamine, phenylephrine, milrinone, and dobutamine within the first two days of ICU admission.

**Figure S1. Restricted cubic spline analyses of the association of SHR with short-term mortality**

1. **(B)**


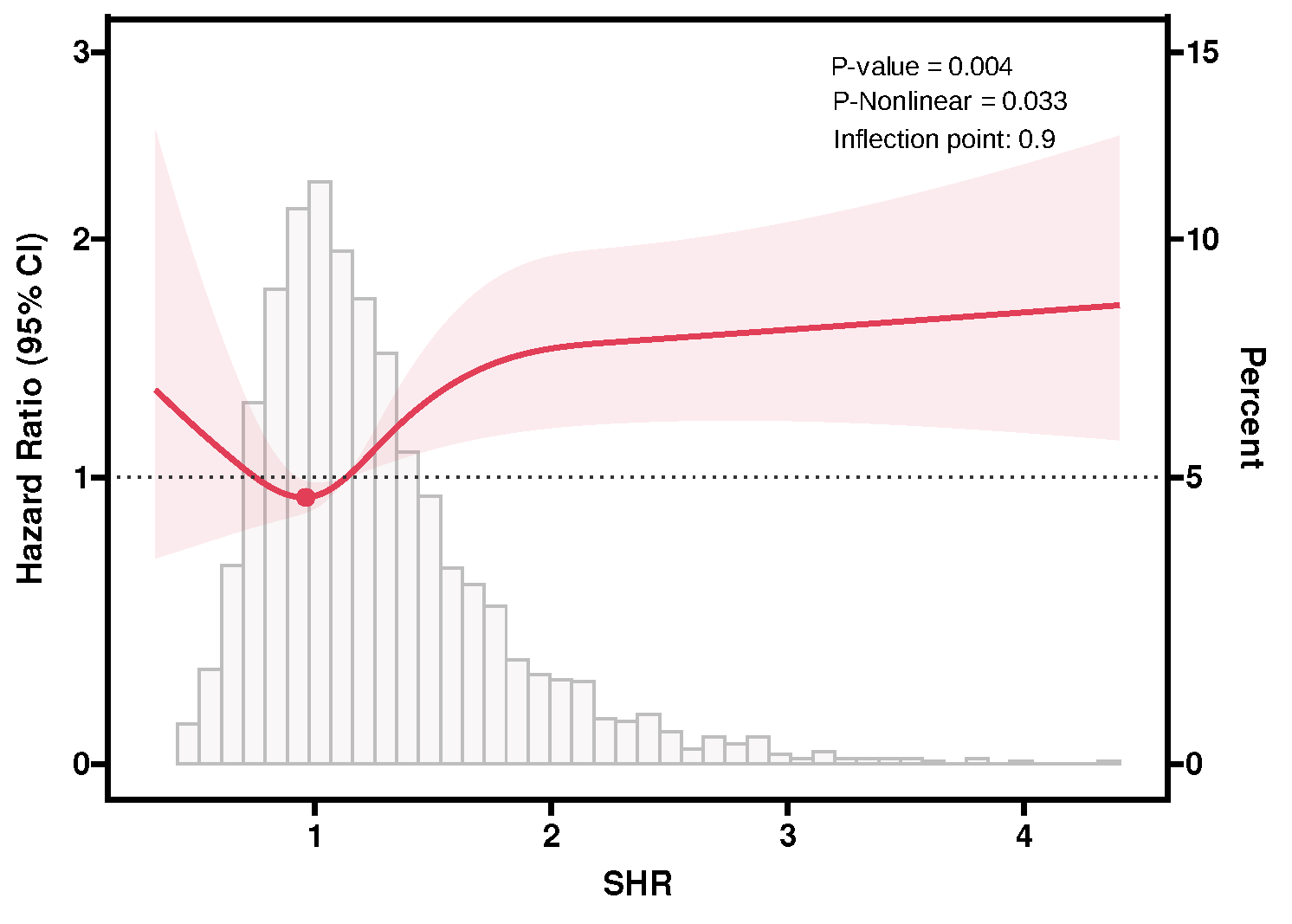

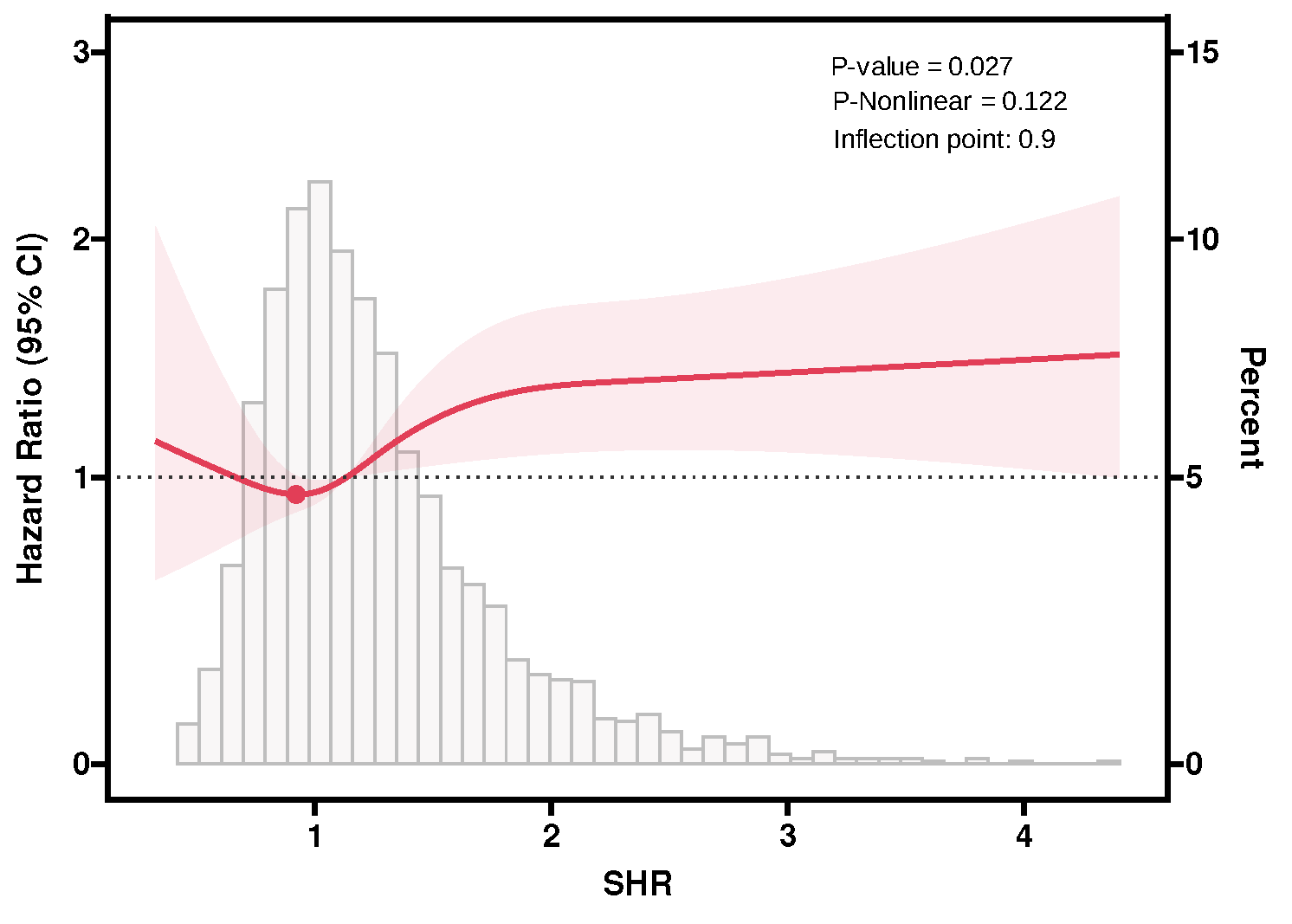


**(C)**

**
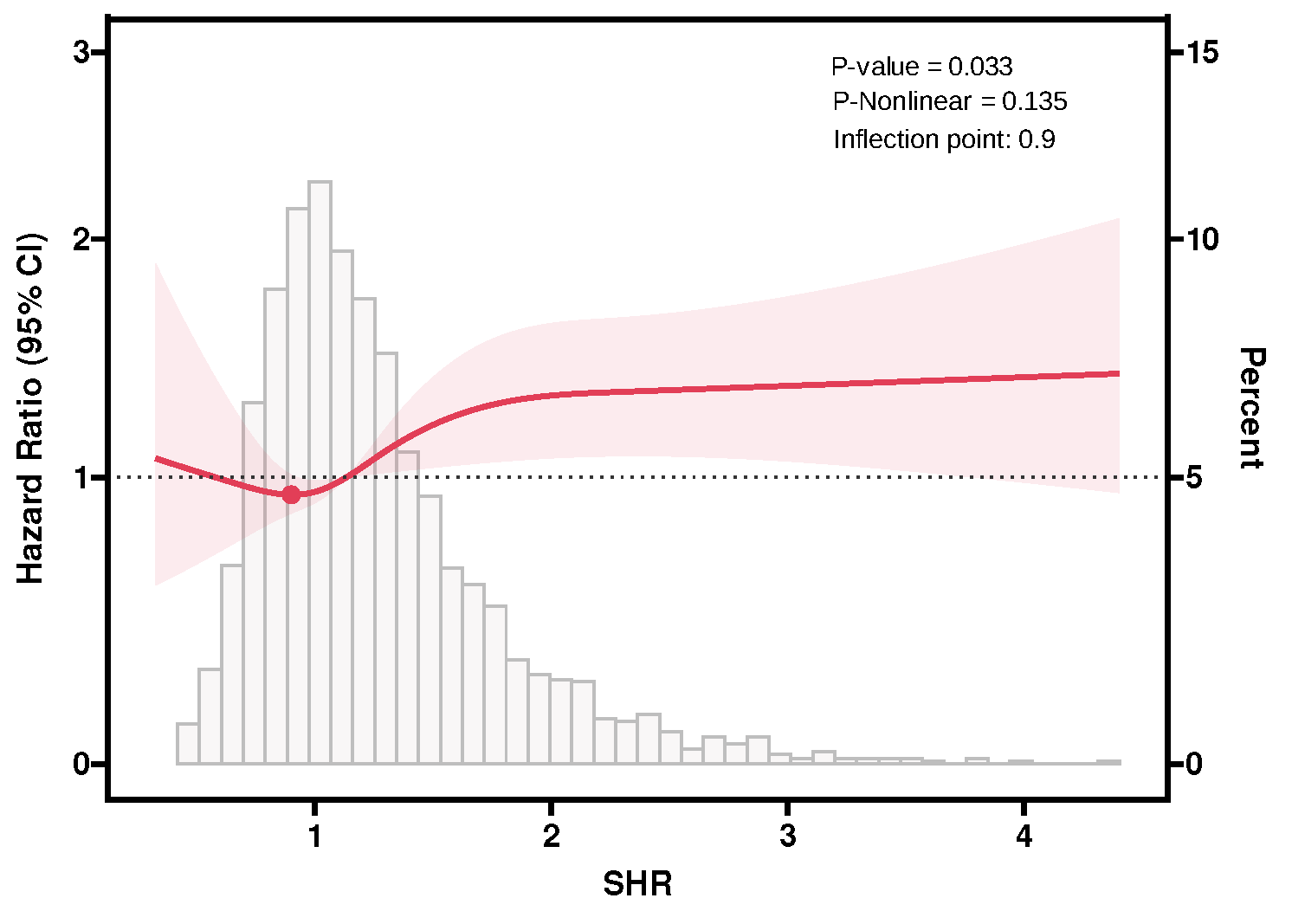
**

Restricted cubic spline analyses of the association of SHR with short-term mortality(A.28-day mortality, B.60-day mortality C.90-day mortality). Heavy central lines represent the estimated adjusted hazard ratios. The 95% confidence interval is represented by the red band. The adjustment strategy is the same as the Model III.
